# Expression of HIF-1alpha and VEGF in colorectal cancer: association with clinical outcomes and prognostic implications

**DOI:** 10.1186/1471-2407-9-432

**Published:** 2009-12-10

**Authors:** Dan Cao, Mei Hou, Yong-song Guan, Ming Jiang, Yu Yang, Hong-feng Gou

**Affiliations:** 1 Department of Medical Oncology, Cancer Center of West China Hospital, Sichuan University, Chengdu, China

## Abstract

**Background:**

Hypoxia-inducible factor 1 alpha (HIF-1α) and vascular endothelial growth factor (VEGF) are frequently overexpressed in numerous types of cancers and are known to be important regulators of angiogenesis. Until now, few studies have been carried out to investigate the prognostic role of these factors in solid tumors, especially in colorectal cancer (CRC). The purpose of this study was to evaluate the expression of HIF-1α and VEGF in CRC tissues, and to analyze the association of these two factors with several clinical and pathological characteristics, and patients' survival.

**Methods:**

Paraffin-embedded tissue samples were retrospectively collected from 71 CRC patients, who received surgical resection between 2001 and 2002, with a median follow-up of 5 years. We examined the patterns of expression of HIF-1α and VEGF by immunohistochemistry method. Statistical analysis was performed with univariate tests and multivariate Cox proportional hazards model to evaluate the differences.

**Results:**

Expression of HIF-1α and VEGF was positively observed in 54.93% and 56.34% among the patients, respectively. HIF-1α and VEGF status were significantly associated with tumor stage, lymph nodes and liver metastases (*P *< 0.05). Expression of both HIF-1α and VEGF remained significantly associated with overall survival (OS) (*P *< 0.01), and HIF-1α was positively correlative to VEGF in CRC (r = 0.72, *P *< 0.001).

**Conclusions:**

HIF-1α and VEGF could be used as biomarkers indicating tumors in advanced stage and independently implied poor prognosis in patients with CRC. Treatment that inhibits HIF-1α might be a promising targeted approach in CRC to exhibit its potential to improve outcomes in future perspective, just as VEGF targeting has proved to be.

## Background

Colorectal cancer (CRC) still accounts for a high morbidity and mortality, worldwide, in men and women [1]. Earlier diagnosis, better acknowledge of its clinical prognostic factors and surgical treatment combined with chemotherapy or radiotherapy had contributed to the improved efficacy and clinical outcomes of the affected patients. Significant improvements have been made in the management of this disease mainly through the administration of active chemotherapeutic agents, involving oxaliplatin and irinotecan, combined with fluorouracil [2,3]. In recent years, advances in understanding tumor biology have led to the development of targeted therapies making progress in the treatment of CRC [4-6]. The most significant and independent prognostic factors accepted to date in CRC remain the TNM (tumor-node-metastasis) stage according to the initial surgery [7]. Moreover several other factors providing predictive value for survival have been identified, but only a few of them have shown to have an impact on CRC patients' prognosis and survival including 'potential' residual disease, obstruction, histology perforation, venous invasion and inadequate number of lymph nodes evaluated [8]. In fact, some prognostic factors are still unknown. Although TNM classification is useful for staging and selecting patients for specific treatment, this classification is insufficient as many patients at the same stage may have various outcomes. Hence, additional prognostic biomarkers are really needed for the management of CRC patients nowadays.

Insufficient levels of cellular O_2_, a condition known as hypoxia, were demonstrated in pathological states due to rapid proliferation of tumor cells. Hypoxia exists in microenvironment in many tumor entities due to structural and functional abnormality of vessels and increasing oxygen consumption caused by rapid proliferation of tumor cells. Hypoxia inducible factor-1 (HIF-1), a transcriptional complex, which generally resides in anoxic mammal and human cells, has already been identified as one critical protein directly reacting to hypoxia [9]. No tumors grow larger than 2 mm^3 ^in the absence of angiogenesis because lack of oxygen in the center of the tumor results in cell apoptosis and necrosis. Reduced oxygen tension (hypoxia) is a key signal for the induction of angiogenesis, and one of the key angiogenic factors regulated by hypoxia is VEGF [10]. All these factors are under the control of HIF-1 which specially binds to the hypoxia response element (HRE) that includes cis-acting DNA elements recognized by multiple transactivators [11]. HIF-1 is a heterodimer composed of HIF-1alpha (HIF-1α) and HIF-1beta (HIF-1β) subunits. HIF-1β, also known as aryl hydrocarbon receptor nuclear translocator, is constitutively expressed, whereas HIF-1α activates the expression of VEGF gene by binding to the HRE in the VEGF promoter region [12,13].

Above all, HIF-1α and VEGF are both important regulators of angiogenesis. Understanding association of tumour biology with clinical features is crucial for the development of antiangiogenic therapy. Although HIF-1α and VEGF were reported to be expressed in many types of cancers, few studies which investigate the clinical value of these factors in solid tumors, in particular, in human CRC, have yet been reported. The purpose of our study was to investigate the impact of HIF-1α and VEGF expression on clinical outcomes and prognosis in human CRC. Moreover, we have demonstrated the interaction between these two factors.

## Methods

### Patients

Eighty paraffin-embedded tumor samples were available from 80 consecutive patients diagnosed in our hospital with CRC between January 2001 and March 2002. Two patients were excluded who had only simple biopsies due to loco-regional extension and seven patients were lost to follow up. Finally, 71 patients were evaluated in the clinical and histological study and for the survival analysis. Samples of normal tissue retrieved up to 5 centimeters away from the tumor's edge were taken as negative controls. Forty-three out of 71 patients were male. The median age was 56.2 ± 10.5 (29-75). No patients had history of chemotherapy or radiotherapy before surgery. The present study involving the human tissue samples was approved by the medical ethics committee of West China Hospital of Sichuan University. Informed consents from the patients were obtained for study of the resection specimens.

### Histology

Fresh CRC samples were received after resection, fixed in 10% pH-neutral formalin, embedded in paraffin. All the patients had the diagnosis of adenocarcinomas and were staged according to the American Joint Commission for Cancer staging (AJCC/TNM, the sixth version) system. Clinicopathological characteristics in our study included age, gender, tumor size, degree of histological differentiation (well/moderate/poor, WHO), depth of infiltration, staging, and status of lymph nodes and liver metastases. All histological slides were reviewed by two senior pathologists from our institution to confirm the diagnoses, and to evaluate the patterns and intensity of HIF-1α and VEGF reactivity.

### Immunohistochemistry

**Figure 1:**
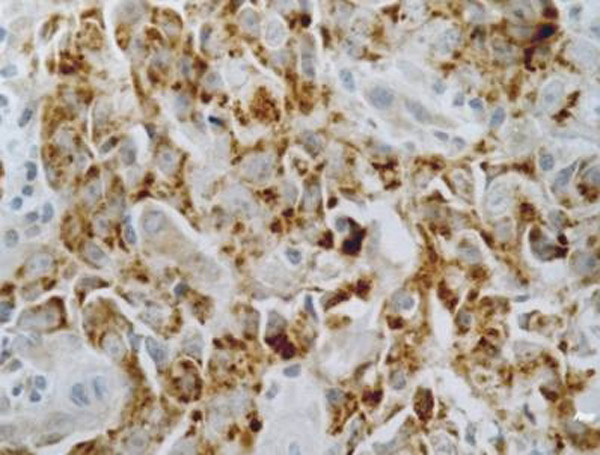
**Positive control of the patterns of HIF-1α expression**. Samples known to express HIF-1α intensely served as the positive control repoted by Zhong.

**Figure 2:**
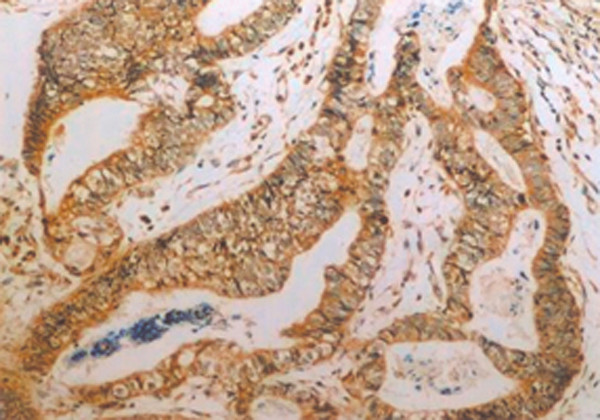
**Positive control of VEGF expression**. Samples used as the positive control of VEGF expression reported by Shibusa.

Immunohistochemistry was performed using the un-avidin-biotin complex technique named EnVision following the manufacturer's instructions with the reagents supplied together with the kit. Briefly, 4 μm-thick sections were cut consecutively from formalin-fixed, paraffin-embedded tissue. Sections were mounted on silanized slides and allowed to dry overnight at 37°C. After deparaffinization and rehydratation, slides were incubated with 3% hydrogen peroxide solution for 5 min. After a washing procedure with the supplied buffer, tissue sections were repaired for 40 min with ethylenediamine tetraacetic acid. The slides were again incubated with the primary antibody for 60 min at 37°C and then overnight at 4°C. The primary polyclonal rabbit antibody (HIF-1α, Zymed) and monoclonal mouse antibody (VEGF, DakoCytomation) were both diluted at 1:50. After three rinses in buffer, the slides were incubated with the secondary antibodies (unbiotinylated antibody, EnVisionTM System, HRP, anti-mouse/rabbit, DakoCytomation). Tissue staining was visualized with a DAB substrate chromogen solution (DakoCytomation). Slides were counterstained with hematoxylin, dehydrated, and mounted. Each run included, for each patient, phosphate buffered solution (PBS) used as the primary antibody for the negative controls, while samples known to express HIF-1α and VEGF strongly served as the positive controls (Figure 1,2).

### Scoring criteria

Five fields of vision by high power lens (×400) were selected randomly and 200 cells were counted per field. Then the percentage of positive cells was calculated. As reported by others, HIF-1α cores were scored according to the presence of nuclear and cytoplasmic staining [14]. Because the level of HIF-1α that is required to initiate transcription is currently unknown, and the range of staining intensity was narrow, the HIF-1α cores were scored only according to the presence (1+) or absence (0) of nuclear and cytoplasmic expression. Positive expression was defined by staining of more than 1% of the tumor area. Cytoplasmic immunoreactivity for VEGF was evaluated according to the extent of staining, with staining intensities scored as: 0, no cells stained; 1, less than 10% of cells stained; 2, 11% to 50% of cells stained; 3, more than 50% of cells stained. For statistical analysis, we divided the patients into two groups, positive expression being defined by observation of staining of more than 10% of the tumor area (scores 2-3) [15].

### Statistical analyses

Statistical analyses were all performed using SPSS-11.5 software. To test for difference between positive and negative of HIF-1α and VEGF expression scores, the chi-square analysis was performed for categorical variables. Various typical factors were considered for univariate analysis. The impact of HIF-1α and VEGF expression on survival was assessed with the Kaplan-Meier method and compared by the log-rank test. Multivariate analysis, conducted with a backward stepwise application of Cox regression was used to evaluate the influence of HIF-1α and VEGF expression on survival. Spearman correlation from ranks was used to analyze the interaction between HIF-1α and VEGF. The results were defined as *P *≤ 0.05 for statistical significance.

## Results

### Patient characteristics

Of the 71 tumor tissues obtained, 31 cases (43%) located in the sigmoid colon. The majority (84%) was well and moderately differentiated adenocarcinoma. Of the all patients at the time of diagnosis, 27 had lymph nodes metastases and 11 had liver metastases. During the referred range of years, 2001-2006, after resection, 42 patients received chemotherapy according to their stage III or IV. The adjuvant chemotherapy for patients with stage III CRC in our institution was a 5-fluorouracil (5 FU)/leucovorin-based regimen or with oxaliplatin. In metastatic CRC, patients received either 5 FU with leucovorin in combination with oxaliplatin or irinotecan. However, no patients received such new therapeutic strategy as HIF-1α and VEGF targeted therapy because targeting drugs of this kind had not been prescribed.

### HIF-1alpha and VEGF patterns of expression

**Figure 3:**
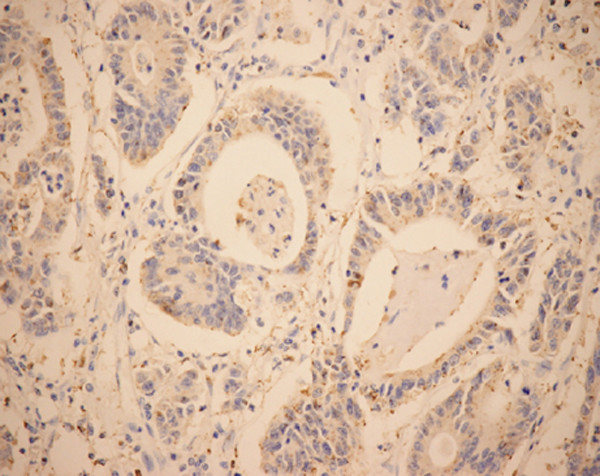
**Immunohistochemical staining of HIF-1α in moderately differentiated colon adenocarcinomas (EnVision, ×200)**. Typical mixed nuclear/cytoplasmic immunostaining of HIF-1α.

**Figure 4:**
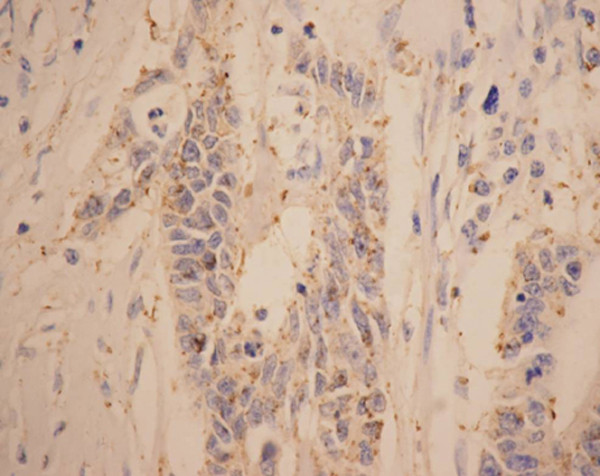
**Staining of immunohistochemistry of HIF-1α (EnVision, ×400)**. HIF-1α expression showing mixed nuclear and cytoplasmic staining.

**Figure 5:**
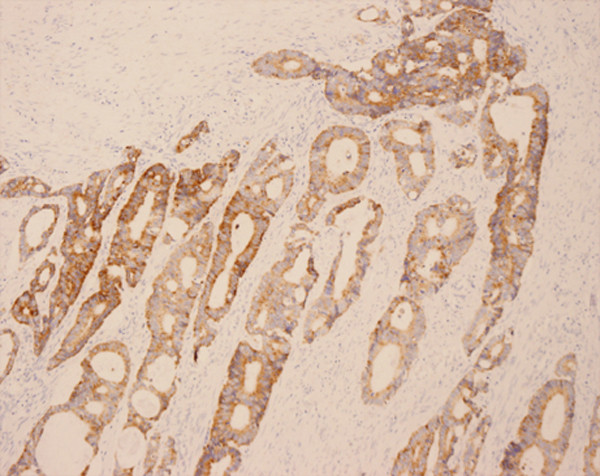
**Immunohistochemical staining of VEGF in colorectal cancer (EnVision, ×200)**. VEGF expression showing a strong cytoplasmic immunostaining.

**Figure 6:**
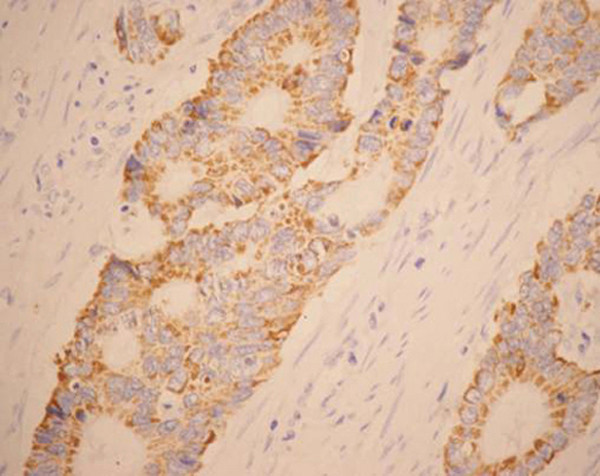
**Staining of VEGF in colorectal cancer cells (EnVision, ×400)**. Typical cytoplasmic immunoreactivity of VEGF.

The patterns of HIF-1α expression in tumor tissues, when present, were mixed nuclear/cytoplasmic (Figure 3) and HIF-1α was positive in 54.93%(39/71) of the patients (Figure 4). HIF-1α protein was expressed strongly in the epithelium around the tumor, especially in the necrosis region, but not in normal mucosa. VEGF was positive in 56.34% of the tumors (Figure 5, 6) and 17.39% of normal mucosa.

### Relationship with clinical outcomes

In univariate analysis, neither the patterns of HIF-1α expression nor VEGF expression was associated with following clinical parameters: age, sex, tumor size, degree of histological differentiation, and depth of infiltration (P > 0.05). The difference of HIF-1α expression in different stage, lymph nodes and liver metastases had statistical significance (P < 0.001) (Table 1). Overexpression of HIF-1α was found in tissue of stage III and stage IV, lymph nodes and liver metastases. The difference of VEGF expression in different stage, lymph nodes and liver metastases had statistical significance (P < 0.001) (Table 2).

**Table 1:** Association between HIF-1α and clinicopathological factors

Factors	No. of patients	HIF-1α		
	
		Positive expression	χ^2^	*P*
Age				
<60	24	15(62.5%)	0.839	0.363
≥ 60	47	24(51.1%)		
Sex				
Male	43	24(55.8%)	0.034	0.853
Female	28	15(53.6%)		
Tumor size				
<5 cm	31	17(54.8%)	0.000	0.989
≥ 5 cm	40	22(55%)		
Differentiation grade				
Poor	11	4(36.4%)	2.233	0.327
Moderate	48	27(56.3%)		
Well	12	8(66.7%)		
Invasion Depth				
T1 + T2	10	6(60%)	0.121	0.728
T3 + T4	61	33(54.1%)		
Tumor stage				
I+II	42	15(35.7%)	15.336	<0.001
III+IV	29	24(82.8%)		
lymphnode matastasis,				
No	44	17(38.6%)	12.007	<0.001
Yes	27	22(81.5%)		
liver matastasis				
NO	60	29(48.3%)	6.806	0.009
Yes	11	10(90.1%)		

**Table 2:** Relationship between VEGF and clinicohistological features

Factors	No. of patients	VEGF		
	
		Positive expression	χ^2^	*P*
Age				
<60	24	14(58.3%)	0.059	0.809
≥ 60	47	26(55.3%)		
Sex				
Male	43	25(58.1%)	0.144	0.704
Female	28	15(53.6%)		
Tumor size				
<5 cm	31	19(61.3%)	0.549	0.459
≥ 5 cm	40	21(52.5%)		
Differentiation grade				
Poor	11	4(36.4%)	5.431	0.066
Moderate	48	26(54.2%)		
Well	12	10(83.3%)		
Invasion Depth				
T1 + T2	10	5(50%)	0.190	0.663
T3 + T4	61	35(57.4%)		
Tumor stage				
I+II	42	12(28.6%)	32.229	<0.001
III+IV	29	28(96.6%)		
lymphnode matastasis,				
No	44	14(31.8%)	28.280	<0.001
Yes	27	26(96.3%)		
Liver matastasis				
NO	60	30(50%)	6.324	0.012
Yes	11	10(90.9%)		

In multivariate analysis, logistic regression retained a significant association between HIF-1α expression and tumor stage (*P *= 0.001), with the percentage of patients whose tumors expressed HIF-1α being 8.640-fold higher in TNM stage III + IV than TNM stage I + II (regression coefficient = 2.156, relative risk = 8.640). There was no significant association between VEGF expression and the clinicopathological factors.

### Impact of HIF-1α and VEGF expression on prognosis

#### Univariate prognostic analyses

**Figure 7:**
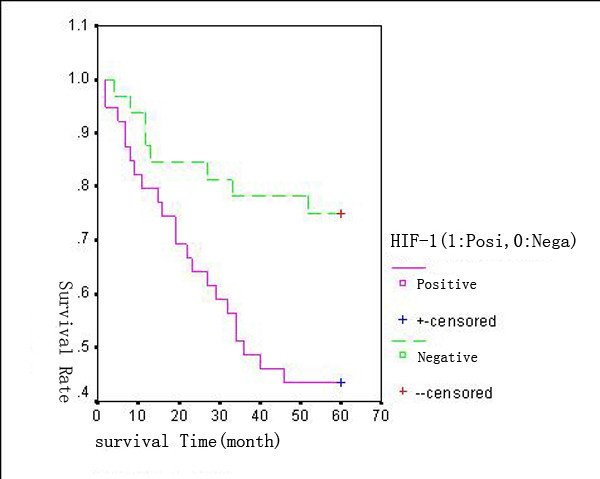
**Kaplan--Meier overall survival (OS) curves for 71 patients with colorectal cancer**. Patients with HIF-1α positive expression had a significantly worse OS compared with those with HIF-1α negative expression.

**Figure 8:**
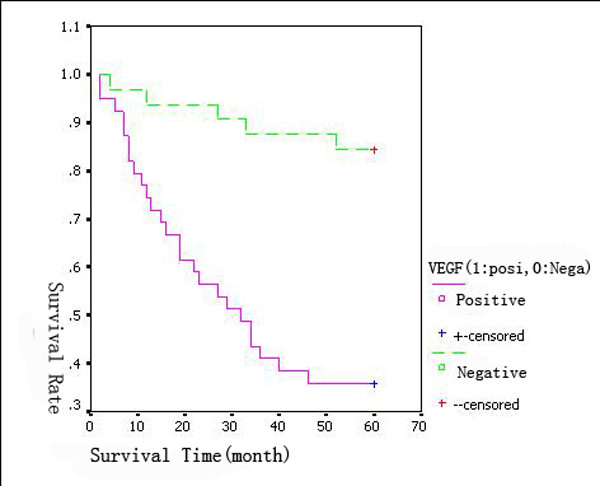
**Impact of VEGF expression on the patients' overall survival (log-rank test)**. The survival time of patients with VEGF positive expression was significantly shorter than that of patients with negative expression.

With a total follow-up of 60 months, 42 of the 71 assessable patients were still alive and 29 patients were known to have died. The 5-years overall survival (OS) for all patients was 59.15%. Analysis of the impact of negative or positive HIF-1α expression composite score on OS is shown in Figure 7. The 5-years OS for patients with negative and positive HIF-1α expression was 75% and 46% respectively. Patients with HIF-1α positive tumors tended to have poorer prognosis than did patients with negative tumors. The expression of HIF-1α was negatively associated with the survival time of patients (P < 0.01) (log-rank test). The 5-years OS of patients with negative and positive VEGF expression was 83.87%(26/31) and 40% (16/40). The survival time of patients with VEGF positive expression was significantly shorter than that of patients with VEGF negative expression (P < 0.01) (Figure 8).

#### Multivariate analysis and Cox's proportional hazard model

According to our multivariate analysis (Cox model), using all the influential factor variables HIF-1α and VEGF, HIF-1α expression (*P *= 0.02) remained significantly associated with OS (RR = 2.69, 95% confidence interval, 1.15-6.30) and VEGF expression (*P *= 0.81) had no significant association with prognosis (RR = 1.13, 95%CI, 0.41-3.09). There was a trend toward poorer survival among the patients of VEGF positive expression. Multivariate analysis identified that HIF-1α was statistically significant as an independent prognostic factor for OS.

### Correlation of HIF-1alpha and VEGF expression in colorectal cancer

Of the 39 patients with positive HIF-1α expression, 34 patients were investigated with positive VEGF expression. Positive expression of VEGF was detected in 34 patients. While of the 32 patients with negative HIF-1α expression, 27 patients were evaluated with negative VEGF expression. Negative expression of VEGF was evaluated in 27 patients. Spearman analysis showed the expression of HIF-1α was positively correlative to VEGF (r = 0.716, P < 0.001) in CRC (Table 3).

**Table 3:** Correlation between HIF-1α and VEGF in CRC

		VEGF
HIF-1α	*r*	0.716
	*P*	<0.001

## Discussion

The main findings of the present study are that HIF-1α and VEGF expression were significantly associated with advanced stage and implied poor prognosis in patients with CRC. Moreover, in multivariate analysis overexpression of HIF-1α was an independent prognostic factor for OS.

As we know, HIF-1 and VEGF are important regulators of angiogenesis [16]. HIF-1 is a heterodimer composed of HIF-1α and HIF-1β subunits. HIF-1β also known as aryl hydrocarbon receptor nuclear translocator, is constitutively expressed, whereas HIF-1α is protected from ubiquitination and proteasomal degradation under hypoxic conditions [17]. HIF-1α activates transcription of genes encoding glucose transporters, glycolytic enzymes, and VEGF. HIF-1α activates the expression of VEGF gene by binding to the HRE in the VEGF promoter region [18]. Tumor angiogenesis is stimulated by angiogenic inducers involving VEGF, basic fibroblast growth factor (bFGF), transforming growth factor (TGF) and interleukin 8 (IL-8) [19]. HIF-1α and VEGF are major regulators of angiogenesis [20] and are important in tumor progression [21].

This study was performed to evaluate retrospectively HIF-1α and VEGF immunohistochemical reactivity in CRC patients and to explore the association of the expression with clinicopathological characteristics and prognosis. HIF-1α was found to be overexpressed in 13 of 19 tumor types compared with the respective normal tissues, including colon, breast, gastric, lung, skin, ovarian, pancreatic, prostate, and renal carcinomas [22-25]. HIF-1α was overexpressed in primary and metastatic human tumors [26-30]. Our study shows that HIF-1α expression was positive in 54.93%(39/71) of CRC but negative in normal mucosa. The tumor cells surrounding necrotic regions expressed high levels of HIF-1αprotein. VEGF was positive in 56.34% of tumors and 17.39% of normal mucosa. The levels of expression of HIF-1α and VEGF in tumor cells were both higher than those in normal cells.

Through the research, we have found that the expression of HIF-1α in different age, sex, tumor size, degree of histological differentiation, and depth of infiltration had no difference(P > 0.05). Consistent with the results of the major studies published to date [31,32], the difference of HIF-1α expression in different stage, lymph nodes and liver metastases had statistical significance(*P *< 0.001). Overexpression of HIF-1α was found in tissue of stage III + IV, lymph nodes and liver metastases. In multivariate analysis, logistic regression confirmed a significant association between HIF-1α expression and tumor stage. In univariate analyses, VEGF expression was significantly associated with different stage, lymph nodes and liver metastases(*P *< 0.001). Initially concluded, the positive expression of HIF-1α and VEGF was significantly associated with advanced stage and seemed to reflect more aggressive histological and clinicalbehaviors. HIF-1α and VEGF could be biomarkers indicating tumor infiltration and metastasis evaluation in CRC.

Previous studies suggest that HIF-1α positivity was associated with both TNM stage and vascular invasion in rectal cancer [33]. In the present study we focused on the clinical and prognostic implications of the expression of HIF-1α and VEGF in patients with CRC, not only rectal cancer. Our results shows that the 5-years OS of patients with negative and positive HIF-1α expression was 75% and 46%, respectively. Patients with positive HIF-1α tumors had a statistically significant poorer prognosis in comparison with patients with negative HIF-1α tumors (*P *< 0.01). VEGF is a survival factor for tumor endothelium in a marine model of colorectal carcinoma with liver metastases [34]. In our study, the 5-years OS of patients with negative and positive expression of VEGF was 83.87%(26/31) and 40% (16/40). The survival time of patients with VEGF positive expression was significantly shorter than that of patients with negative expression (*P *< 0.01). As a result, the positive expression of HIF-1α and VEGF both implied poor prognosis.

In multivariate analysis of Cox model, HIF-1α was an independent prognostic variable for OS (RR = 2.69). VEGF had a trend to be a risk factor of mortality (RR = 1.129) but no statistically significant association withprognosis. This different finding about VEGF in univariate and multivariate analysis should logically encourage us to continue our investigations and enlarge sample size. The present study reinforces the evidence which implies that positive expression of HIF-1α has poor prognosis, maybe the same with VEGF. This study shows that HIF-1α expression is significantly associated with prognosis of patients in CRC.

HIF-1α expression is evaluated in many human cancers and HIF-1α levels of expression in cells correlate with tumorigenicity and angiogenesis [32]. Spearman analysis shows the expression of HIF-1α was positively correlative to VEGF(r = 0.72, *P *< 0.001) in CRC. However, efforts of further research are required to determine the precise correlation between HIF-1α and VEGF.

## Conclusion

Angiogenesis is an early event in carcinogenesis and plays an important role in tumor growth. In line with the expression of VEGF, one of the key angiogenic factors regulating VEGF is HIF-1α. HIF-1α and VEGF might be used as biomarkers indicating tumor infiltration and poor prognosis in human CRC. Inhibition of HIF-1α might be a promising targeted antiangiogenic therapy in CRC to exhibit its potential to improve outcomes in future perspective, just as VEGF targeting has proved to be.

## Competing interests

The authors declare that they have no competing interests.

## Authors' contributions

DC and MH carried out the design of the study, the patients follow up, immunohistochemistry staining, performed the statistical analysis and drafted the manuscript. YSG helped to write, organize and correct the paper. MJ, YY, and HFG participated in immunohistochemistry stain and the statistical analysis. All authors read and approved the final manuscript.

## Pre-publication history

The pre-publication history for this paper can be accessed here:

http://www.biomedcentral.com/1471-2407/9/432/prepub
